# EGFR copy number alterations in primary tumors, metastatic lymph nodes, and recurrent and multiple primary tumors in oral cavity squamous cell carcinoma

**DOI:** 10.1186/s12885-017-3586-9

**Published:** 2017-08-30

**Authors:** Shiang-Fu Huang, Huei-Tzu Chien, Sou-De Cheng, Wen-Yu Chuang, Chun-Ta Liao, Hung-Ming Wang

**Affiliations:** 1 Department of Otolaryngology, Head and Neck Surgery, Chang Gung Memorial Hospital, No. 5 Fu-Shin Street, Kwei-Shan, Taoyuan, Taiwan; 2 Department of Public Health, Chang Gung University, Tao-Yuan, Taiwan; 3 Taipei CGMH Head and Neck Oncology Group, Tao-Yuan, Taiwan; 4 Department of Nutrition and Health Sciences, Chang Gung University of Science and Technology, Tao-Yuan, Taiwan; 5 Department of Anatomy, Chang Gung University, Tao-Yuan, Taiwan; 6 Department of Pathology, Chang Gung Memorial Hospital, Tao-Yuan, Taiwan; 7 Division of Hematology/Oncology, Department of Internal Medicine, Chang Gung Memorial Hospital, Tao-Yuan, Taiwan

**Keywords:** Epidermal growth factor receptor (EGFR), Gene amplification, Recurrence, metastasis, Multiple primary tumors, fluorescence in situ hybridization, Oral cavity squamous cell carcinoma

## Abstract

**Background:**

The EGFR and downstream signaling pathways play an important role in tumorigenesis in oral squamous cell carcinoma (OSCC). Gene copy number alteration is one mechanism for overexpressing the EGFR protein and was also demonstrated to be related to lymph node metastasis, tumor invasiveness and perineural invasion. Therefore, we hypothesized that EGFR gene copy number alteration in the primary tumor could predict amplification in recurrent tumors, lymph node metastatic foci or secondary primary tumors.

**Methods:**

We recruited a group of newly diagnosed OSCC patients (*n* = 170) between Mar 1997 and Jul 2004. Metastatic lymph nodes were identified from neck dissection specimens (*n* = 57). During follow-up, recurrent lesions (*n* = 41) and secondary primary tumors (SPTs, *n* = 17) were identified and biopsied. The EGFR gene amplifications were evaluated by fluorescence in situ hybridization (FISH) assay in primary tumors, metastatic lymph nodes, recurrences and SPTs.

**Results:**

Of the 170 primary OSCCs, FISH showed low EGFR amplification/polysomy in 19 (11.4%) patients and amplification in 33 (19.8%) patients. EGFR gene amplification was related to lymph node metastasis (χ2 trend test: *p* = 0.018). Of 57 metastatic lymph nodes, nine (15.8%) had EGFR polysomy and 14 (24.6%) had EGFR gene amplification. The concordance rate of EGFR gene copy number in primary tumors and lymph node metastasis was 68.4% (McNemar test: *p* = 0.389). Of 41 recurrent tumors, five (12.2%) had EGFR polysomy and five (12.2%) had gene amplification. The concordance rate of EGFR gene copy number between primary tumors and recurring tumors was 65.9% (McNemar test: *p* = 0.510). The concordance rate between primary tumors and SPTs was 70.6%. EGFR amplification in either primary tumors, metastatic lymph nodes or recurrent tumors had no influence on patient survival.

**Conclusion:**

We can predict two-thirds of the EGFR gene copy number alterations in lymph node metastasis or recurrent tumors from the analysis of primary tumors. For OSCC patients who are unable to provide lymph node or recurrent tumor samples for EGFR gene copy number analysis, examining primary tumors could provide EGFR clonal information in metastatic, recurrent or SPT lesions.

**Electronic supplementary material:**

The online version of this article (10.1186/s12885-017-3586-9) contains supplementary material, which is available to authorized users.

## Background

In Taiwan, oral cancer is the 4th most common cancer in men [1]. The consumption of areca-quid (AQ), tobacco and alcohol among Taiwanese men results in an increase in oral cancer risks about ten-fold higher than women and its incidence is rising [2]. The primary treatment for oral cavity squamous cell carcinoma (OSCC) is radical surgery with or without post-operative adjuvant radio−/chemotherapy and this treatment approach can result in good loco-regional control [3]. Some patients have recurrence and/or distant metastasis after these radical treatments. Among the poor prognostic factors for OSCC discussed by O’Brien et al., cervical lymph node metastasis is just as important as tumor stage, the extent of the tumor invasion, and perineural/lympho-vascular invasion in adversely influencing tumor control [4–6]. We previously demonstrated that lymph node metastasis, tumor cell differentiation and perineural invasion and tumor stage are correlated with EGFR gene amplification [7]. Those previous findings indicate that tumor cells with EGFR amplification are invasive. These tumor cells are more likely to proliferate in recurrent tumors and metastasize to the lymph nodes. It is therefore worthwhile to investigate tumor cells with increased EGFR amplification because the number of EGFR copies plays an important role in metastasis, recurrence or development of secondary primary tumors (SPTs).

Regarding the concordance between the number of EGFR gene copies and primary tumor and metastatic lesions in non-small cell lung cancer, the discordant rate ranges from 27 to 32% [8–11]. Due to mucosal “field cancerization”, OSCC patients carry a higher risk of developing SPTs in their head and neck region [12, 13]. The genetic alterations between the primary lesion and secondary primaries are more complex and reflected in markers such as TP53, microsatellite markers or the D-loop region in mitochondria [14, 15]. However, the concordance rate varies depending on the markers used. Our aim in this study was therefore to determine the clonality of EGFR from the primary tumor, metastatic lesion, recurrence and SPT lesions in OSCCs.

We hypothesized that the number of EGFR gene copy alterations in the primary tumor can predict whether tumors will reoccur or whether patients will be at risk for lymph node metastasis. Current knowledge how tumor cells with EGFR gene copy number alterations in the primary tumor are related to metastases and recurrences in OSCC is limited. More specifically, until now no investigations had been conducted in an oral cavity cancer. Therefore, in our study, the status of EGFR gene copy number was investigated in paired samples from a series of primary OSCC lesions and corresponding lymph node metastases, recurrent tumors and even multiple primary tumors. By clarifying the clonality of the EGFR gene status in paired tumor specimens, we can determine whether EGFR amplified cells bear the invasive characters in metastasis or recurrence in oral cavity cancer.

## Methods

### Patients, tissue specimens and clinical diagnosis

This study was approved by the Institutional Review Board of Chang Gung Memorial Hospital. One hundred and seventy oral cancer patients treated at Chang Gung Memorial Hospital, Lin-Kuo, were recruited for participation in this study. All patients gave informed consent for participation and were interviewed uniformly before surgery by a well-trained interviewer. The questionnaire used in the interview sought detailed information on general demographic data, current and past cigarette smoking, alcohol consumption, areca-quid (AQ) chewing, and a history of family disease (Additional file 1). All patients received curative intent surgery as an initial treatment. In the surgeries, the primary tumors were excised with safety margins greater than or equal to 1 cm (for both peripheral and deep margins). The tumor margin tissue was cryosectioned to ensure that the margin was free of tumor. For each patient, clinical histological parameters were scored according to the recommendations for the reporting of specimens containing oral cavity and oropharynx neoplasms by the Association of Directors of Anatomic and Surgical Pathology (ADASP) [16].

### Metastatic lymph nodes

For patients who received radical surgeries, neck dissection was performed according to the tumor stage of the patients. Two types of neck dissections were used in our patients: one was a dissection of level I-III lymph nodes (supraomohyoid neck dissection) for nodal negative patients; and the other was a dissection of level I-V lymph nodes (usually a modified radical neck dissection) for nodal positive patients. We selected pathologically proven metastatic lymph nodes from the neck dissection specimens.

Patients with advanced tumor status (T3 or T4), lymph node extracapsular spread, tumor depth ≥ 10 mm or poor differentiation, adjuvant radiotherapy or cisplatin-based concomitant chemoradiotherapy would be given after surgery.

### Recurrence and secondary primary lesions

After radical surgeries with or without adjuvant chemo-radiotherapy, the patients received regular follow-up visits. For tumors growing nearby the primary tumor, in the neck or distant sites, the lesions were recorded as recurrences. In the head and neck region, the mucosa carries similar risks for developing malignancies. Lesions that were located in different tumor subsites from the primary tumor or a 2 cm distance from the primary lesion in the mucosa were recorded as secondary primary lesions [17]. The secondary lesions could occur simultaneously with the primary lesion (synchronous) or be found during regular follow-up appointments in the clinic after surgeries (metachronous).

### FISH assay and analysis

*EGFR* gene copies were investigated with FISH using the LSI *EGFR* SpectrumOrange/CEP 7 SpectrumGreen probe (Vysis; Abbott Laboratories, Downers Grove, IL) according to the manufacturer’s instructions and our previous report [7]. In brief, section slides were incubated at 56 °C overnight, deparaffinized, dehydrated, treated with 0.2 N HCl (pH 2.5) for 20 min, and treated with 1 M sodium thiocyanate (Sigma-Aldrich Corp., St. Louis, MO) in 1 M Tris (pH 8.0) at 82 °C for 20 min. Then the specimens were digested with 0.4% pepsin (Sigma-Aldrich Corp., St. Louis, MO) in 0.9% NaCl (pH 2.35) for 15 min. The samples were briefly rinsed in ddH_2_O and 2 × SSC between steps. After fixation in 4% formaldehyde for 5 min, each slide had the probe set applied to a selected area, and the hybridization area was covered with a plastic coverslip and sealed with a glue gun before the slides were heated at 75 °C for 10 min with OmniGene (Hybaid Ltd., Middlesex, United Kingdom) to promote co-denaturation of chromosomal and probe DNAs. Hybridization was carried out in a humidified oven at 37 °C for 18 h, followed by post-washing in 0.3% Nonidet P40 (BDH, England) in 2 × SSC at 45 °C for 4 min, in 2 × SSC at 45 °C for 5 min, and finally twice in 2 × SSC at room temperature for 5 min. After being counterstained with DAPI for 5 min, the slides were mounted with Vectashield mounting medium (Vector Laboratories, Burlingame, CA) and scored under an fluorescent microscope using a Plan Neofluar 100× objective (Axiophot, Zeiss, Germany) with dual and triple pass filters (Chroma Technology Corp., Rockingham, VT). At least 100 non-overlapping nuclei per case were scored independently by two independent observers who followed strict scoring guidelines and used constant adjustment of the microscope’s focus because signals were located in different focal planes. In each nucleus, the number of *EGFR* copies and chromosome 7 probes were assessed independently.

**Fig. 1:**
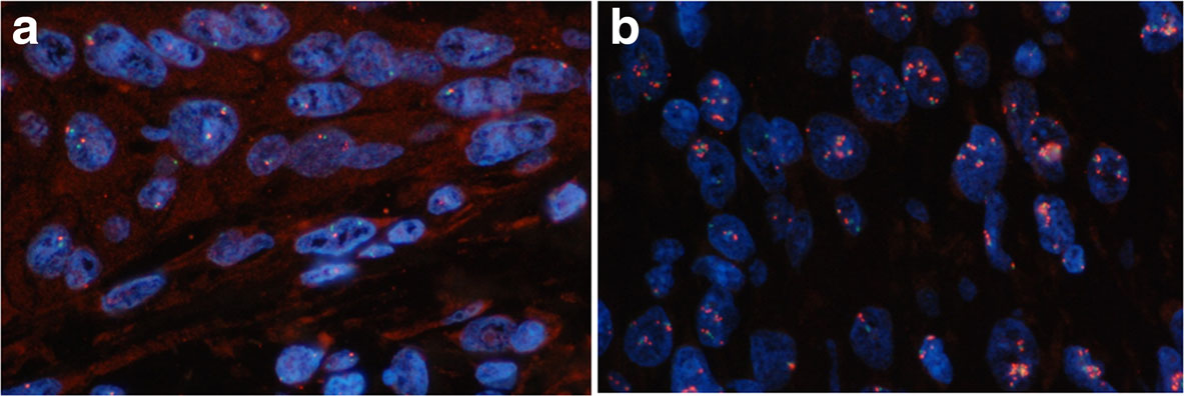
*EGFR* FISH studies in tumor cells. The fields were observed using a triple band filter (630×). **a** Tumor cells with disomy (*EGFR*, SpectrumOrange, Centromere 7 SpectrumGreen). **b** Tumor with EGFR amplifications

FISH patterns were classified into 3 strata based on the number of copies of the *EGFR* gene per cell as described in previous studies [7, 18, 19]. The strata were normal disomy, ≤ two copies in more than 90% of analyzed cells (Fig. 1a); and low amplification/polysomy (LA/Poly), ≥ three copies in more than 40% of analyzed cells. Gene amplification was defined as the presence of tight *EGFR* gene clusters, a ratio of gene/chromosome per cell ≥2, or ≥15 copies of *EGFR* per cell in ≥ 10% of analyzed cells (Fig. 1b). Tumors with LA/Poly or gene amplification were considered to be FISH positive.

### Statistical analysis

Statistical analysis was performed using the SPSS statistical package (SPSS, Chicago, IL). Correlations between the frequency of *EGFR* FISH status and age, TNM stage, cigarette smoking, alcohol consumption, and AQ chewing were examined with the χ^2^ test or Fisher’s exact test. The concordance of EGFR gene copy alterations between primary tumors, metastatic lesions, recurrences and SPTs was analyzed with the McNemar test. Disease-free survival (DFS) was defined as the time from diagnosis to recurrence or metastasis. Overall survival (OS) was defined as the time from diagnosis to death. Survival curves were constructed using the Kaplan-Meier method, and the curves were compared using the log-rank test. A two-sided value of *p* < 0.05 was considered to be statistically significant.

## Results

### Patient characteristics

The clinicopathological features of the 170 OSCC male patients between Mar 1997 and Jul 2004 who took part in this study are listed in Table 1. The major primary sites were the bucca (39.4%, 67/170) and the tongue (34.1%, 58/170). Overall, 90.6% (154/170) of the patients were cigarette smokers, 68.2% (116/170) were alcohol drinkers and 91.2% (155/170) were AQ chewers. All 170 patients received surgery as their initial treatment, and 88 (51.8%) and 30 (17.6%) patients underwent additional radiation therapy and chemoradiotherapy, respectively. The median follow-up was 57.50 months.

**Table 1:** Characteristics of the 170 oral cavity squamous cell carcinoma patients

Characteristic	[No. of patients (%)]
Age (yrs)	
Mean	49.55
Range	29.0–78.0
Site of primary tumor [No. of patients (%)]	
Tongue	58 (34.1)
Mouth floor	8 (4.7)
Lip	6 (3.5)
Buccal mucosa	67 (39.4)
Alveolar ridge	19 (11.2)
Hard palate	4 (2.4)
Retromolar trigone	8 (4.7)
Pathologic tumor status	
T1	30 (17.6)
T2	58 (34.1)
T3	20 (11.8)
T4	62 (36.5)
Pathologic N stage	
N0	101 (59.4)
N1	19 (11.2)
N2b	45 (26.5)
N2c	5 (2.9)
Pathologic stage	
Stage I	22 (12.9)
Stage II	32 (18.8)
Stage III	24 (14.1)
Stage IV	92 (54.1)

Of 170 primary OSCCs, FISH results showed EGFR LA/polysomy in 19 (11.4%) patients and amplification in 33 (19.8%) patients. EGFR gene amplification was related to lymph node metastasis (χ2 trend test: *p* = 0.018). Of 57 metastatic lymph nodes, nine (15.8%) had EGFR polysomy and 14 (24.6%) had EGFR gene amplification.

In our patients, 69 had lymph node metastasis identified in neck dissection specimens and 57 positive lymph nodes from neck dissection specimens were available for FISH assays. Fourteen patients had gene copy number amplification (24.6%, 14/57) and nine (15.8%, 9/57) patients had EGFR gene polysomy. During follow-up, 87 patients had recurrence and 41 recurrence tissues were used for analyzing the EGFR gene copy number. Five (12.2%, 5/41) had EGFR gene amplification and five (12.2%, 5/41) had increased gene copy number (LA/polysomy). Twenty-six patients had secondary primary tumors. A total of 17 secondary primary lesions were suitable for FISH analysis. The results showed that two (11.8%, 2/17) had gene amplification and three (17.6%, 3/17) had an increase in gene copy number. The concordance rate of EGFR gene copy number in primary tumors and lymph node metastasis was 68.4% (McNemar test: *p* = 0.389). The concordance rate between primary tumors and recurrence tumors was 65.9% (McNemar test: *p* = 0.510), and the concordance rate between primary tumors and SPTs was 70.6% (Table 2).

**Table 2:** EGFR gene amplification in primary cancer with recurrence, multiple primaries, and neck metastasis

	EGFR gene copies number				
EGFR gene copies	Disomy [n (%)]	Polysomy [n(%)]	Amplification [n (%)]	Discordance	*P* value
Recurrent tumor					
Disomy (*n* = 31)	24 (77.4)	4 (12.9)	3 (9.7)	14/41 (34.1%)	0.261
Polysomy (*n* = 5)	5 (100.0)	0 (0.0)	0 (0.0)		0.510^*^
Amplificaiton (*n* = 5)	2 (40.0)	2 (40.0)	1 (20.0)		
Second primary tumor					
Disomy (*n* = 12)	11 (91.7)	1 (8.3)	0 (0.0)	5/17 (29.4%)	0.264
Polysomy (*n* = 3)	2 (66.7)	1 (33.3)	0 (0.0)		^*^NA
Amplificaiton (*n* = 2)	1 (50.0)	1 (50.0)	0 (0.0)		
Lymph node metastasis					
Disomy (*n* = 34)	25 (73.5)	2 (5.9)	7 (20.6)	18/57 (31.6%)	<0.001
Polysomy (*n* = 9)	3 (33.3)	4 (44.4)	2 (22.2)		^*^0.389
Amplificaiton (*n* = 14)	4 (28.6)	0 (0.0)	10 (71.4)		

^*^McNemar test

In four patients with multiple primary cancers, the concordance rate of EGFR gene copy number was 100% (Table 3). In one patient (No. 2) with multiple recurrences, the EGFR copy number increased in the recurring tumor. The EGFR gene polysomy was maintained in the second recurrence.

**Table 3:** Summary for EGFR gene copy number alterations in multiple primary OSCC patients

	Case	Primary cancer site	EGFR gene copy number	Second cancer site	EGFR gene copy number	Third cancer site	EGFR gene copy number
1	OR147	Left alveolus	Polysomy	Right tongue	Trisomy with Focal amplification	Left tongue	Polysomy
2	OR218	Left alveolus	Disomy	Recurrence	Trisomy or polysomy	2nd recurrence	Polysomy
3	OR276	Left bucca	Disomy	Right hard palate	Disomy	Right alveolus	Polysomy
4	OR295	Right tongue	Disomy	Left tongue	Disomy	Hard palate (3rd primary)	Disomy
5	OR325	Right mouth floor	Disomy	Soft palatal	Disomy	Recur from 2nd primary	Disomy

### Prognostic implications of EGFR gene copy number in metastatic lymph nodes and tumor recurrence

As shown in Table 4, *EGFR* gene amplification was significantly more prevalent in tumors at an advanced stage than tumors at early stages. Younger patients had a higher risk of EGFR gene amplification. Tumors with high levels of tumor invasion, lymph node metastasis, bone invasion and perineural invasion had a significantly higher frequency of *EGFR* gene amplification than tumors without those characteristics. However, *EGFR* gene amplification was not associated with subsites, skin invasion, AQ chewing, cigarette smoking, and alcohol consumption. We analyzed other factors that may predict EGFR gene amplification in metastatic lymph node and found no clinicopathological factors related to amplification.

**Table 4:** The associations between EGFR gene copies and clinicopathological parameters in recurrent tumor (*N* = 41)

	EGFR Gene Copies Number			
	Disomy [N (%)]	Polysomy [N (%)]	Amplification [N (%)]	*p* value
Subsites				
Local (*n* = 31)	22 (71.0)	4 (80.0)	5 (100.0)	0.643
Regional (*n* = 3)	2 (6.5)	1 (20.0)	0 (0.0)	
Distant metastasis (*n* = 7)	7 (22.5)	0 (0.0)	0 (0.0)	
Tumor status				
T null^a^ (*n* = 10)	9 (29.0)	1 (20.0)	0 (0.0)	0.644
Early^b^ (*n* = 30)	21 (67.7)	4 (80.0)	5 (100.0)	
Advanced^c^ (*n* = 1)	1 (3.2)	0 (0.0)	0 (0.0)	
Lymph node metastasis				
Yes (*n* = 12)	10 (32.3)	2 (40.0)	0 (0.0)	0.289
No (*n* = 29)	21 (67.7)	3 (60.0)	5 (100.0)	
Radiation therapy				
Yes (*n* = 29)	22 (71.0)	4 (80.0)	3 (60.0)	0.936
No (*n* = 12)	9 (29.0)	1 (20.0)	2 (40.0)	
Chemotherapy				
Yes (*n* = 7)	7 (22.6)	0 (0.0)	0 (0.0)	0.256
No (*n* = 34)	24 (77.4)	5 (100.0)	5 (100.0)	

^a^no primary tumor recurrence, but with either lymph node or distant metastasis

^b^Early: T1/T2 lesions

^c^Advanced: T3/T4 lesions

**Fig. 2:**
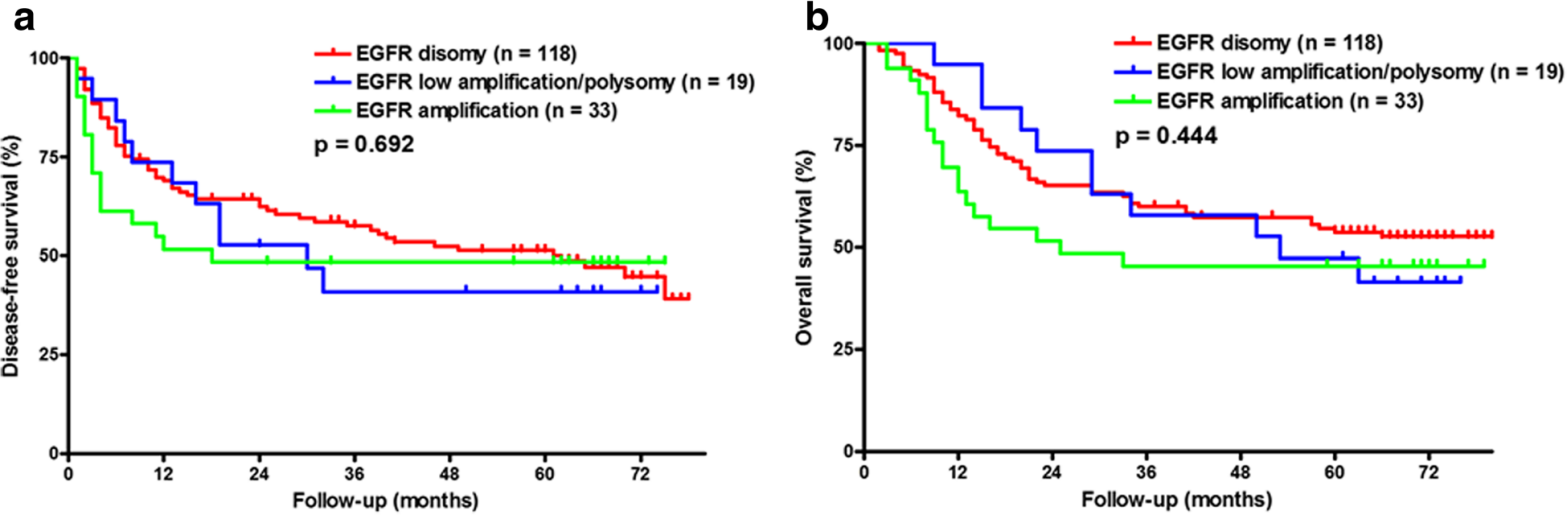
The Kaplan-Meier survival curves for patients with different EGFR gene copy numbers in primary tumors for **a** disease-free survival and **b** overall survival

**Fig. 3:**
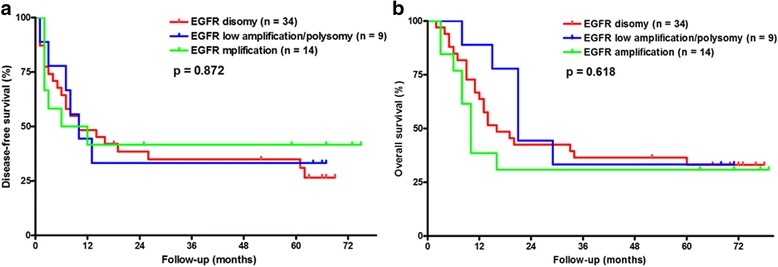
The Kaplan-Meier survival curves for patients with different EGFR gene copy numbers in metastatic lymph nodes for **a** disease-free survival and **b** overall survival

**Fig. 4:**
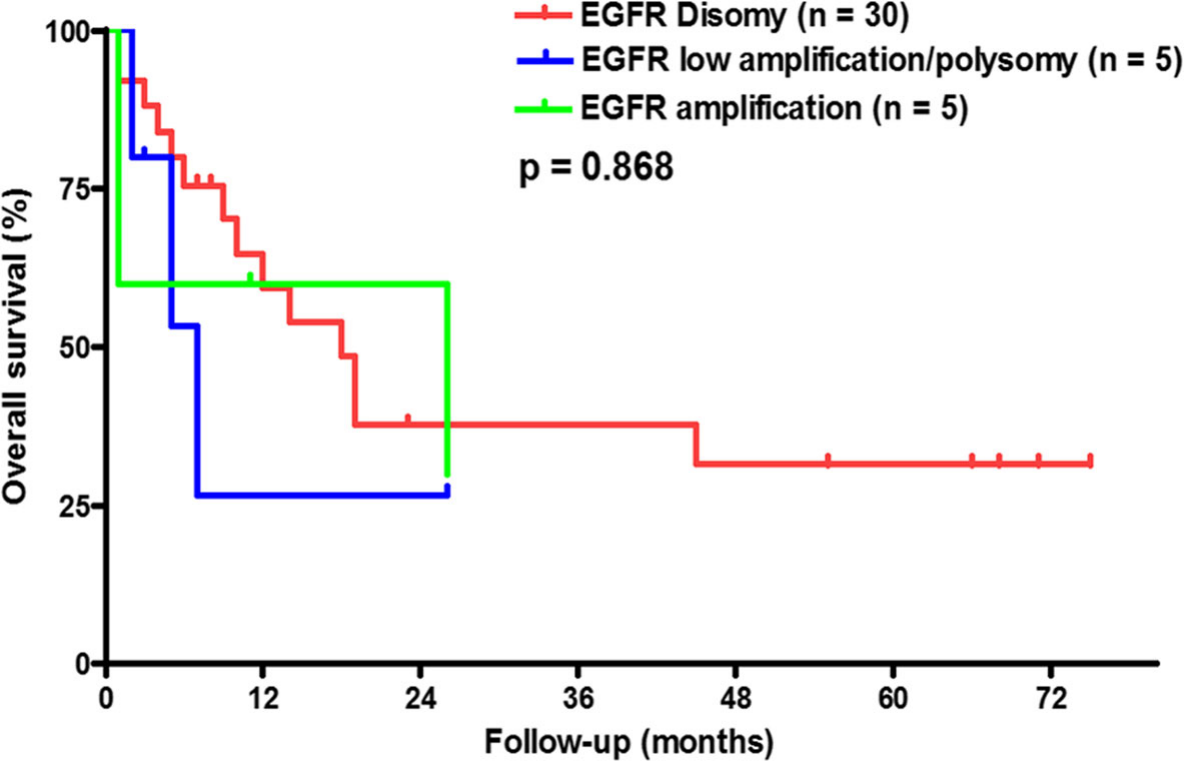
The Kaplan-Meier overall survival curves for patients with different EGFR gene copy numbers in recurrent tumors

The Kaplan-Meier survival curves for patients with different EGFR gene copy numbers are shown in Fig. 2. Patients showing an EGFR FISH pattern were not significantly associated with either DFS or OS (Fig. 2a, *p* = 0.692 and Fig. 2b, *p* = 0.444). The EGFR gene amplification in metastatic lymph nodes was not associated with patient survival (DFS and OS, Fig. 3a, *p* = 0.872, and Fig. 3b, *p* = 0.618, respectively). Furthermore, the EGFR FISH pattern in recurrence tumors did not predict patient survival from recurrence to death (Fig. 4, *p* = 0.868).

## Discussion

For loco-regional advanced head and neck squamous cell carcinoma, concomitant radiotherapy with anti-EGFR target therapy such as Cetuximab (C225, Erbitux™) has been shown to improve locoregional control and reduce mortality [20]. A significant improvement in OS/DFS and response rate were also observed in the EXTREME clinical trial [21]. In non-small cell lung cancer (NSCLC), several reports have also shown that EGFR-specific tyrosine kinase inhibitors, such as gefitinib and erlotinib, are capable of reducing brain and adrenal metastases [22, 23]. EGFR mutations, amplifications or gene gains have been associated with clinical responses to those inhibitors [18, 24]. A previous study demonstrated that *EGFR* FISH analysis may be used as an alternative to gene mutation analysis as the primary laboratory test [25]. Additionally, in our previous study, the EGFR mutation rate in areca-quid-related OSCC was as low as 0.58% [26]. Gene amplification is one of the important mechanisms that influence EGFR proteins expression. We sought to better understand the clonal change of *EGFR gene* between primary tumors, metastatic lesions and recurrence tumors in OSCC.

The tumors examined in our experiments were heterogeneous and polyclonal. Park et al. demonstrated that *EGFR* mutations are not always identical in disseminated cancer cells and cells from the primary tumors in NSCLC [27]. The differences could originate from intratumoral molecular heterogeneity or the consequences of genetic instability during metastatic spread of tumor cells. In our study, we hypothesized that tumor cells with EGFR amplification were prone to recur or metastasize. Our EGFR copy number comparison analyzed primary and metastatic lesions in OSCC, and we found the concordance rate was approximately 60%. In the literature, studies on clonal changes between primary tumors and metastatic lesions in EGFR in OSCC were few and most related studies focused on lung cancer. Matsumoto et al. reported a 100% concordance for EGFR mutation status in six NSCLC patients of Asian ethnicity [28]. In a study by Kalikaki et al., the authors demonstrated significant discordance between EGFR and K-RAS mutations occurring in primary tumors and the corresponding metastases in patients with NSCLC [29]. The discordance in EGFR mutation status was 28% and the discordance for K-RAS was 24%. Similarly, two other studies of paired NSCLC tumors showed discordance rates of 32 and 27% for the EGFR gene copy number [8, 10]. The concordance rate of EGFR copy number in metastatic lymph nodes or recurrent OSCC from our study were within the range of concordance rates for lung cancer.

In a meta-analysis by Wang and Wang, primary NSCLC had a lower EGFR copy number rate (29.3%, 39/133) than corresponding metastases (39.8%, 53/133), but there was no significant difference [30]. In our study, the EGFR copy number in metastatic lymph node samples was higher (40.35%) than samples from primary tumors (30.59%). Although the result was statistically insignificant, tumor cells with EGFR gene amplification carried a higher propensity for lymph node metastasis. In OSCC, an increased EGFR copy number was identified in 24.39% of recurrent tumors and 29.41% of SPTs. We intended to identify the factors that would lead to a higher risk of EGFR gene amplification in patients. In Table 4, no clinical factors, such as primary tumor stage, lymph node metastasis, radiation therapy or chemotherapy, were related to increased EGFR gene copy number. To minimize the heterogeneity of our study population, none had received neo-adjuvant chemotherapy, neoadjuvant bio-chemotherapy or adjuvant bio-CCRT. Adjuvant chemo-radiotherapy for locoregional advanced OSCCs consists of cisplatin-based regimen in our patients. In the patients with tumor recurrence, 70.73% had previous radiation therapy after primary surgery and 17.07% received adjuvant chemotherapy concomitantly with radiation therapy. Interestingly, none of the recurrences had EGFR amplification if the patients had chemotherapy included in the initial treatment of OSCC. The tumor clones of increased EGFR copy number could potentially have been eliminated during the process of recurrence.

## Conclusions

In OSCC, the concordance rates between primary tumors and metastatic lymph nodes, recurrence tumors or SPTs were 65.9, 68.4 and 70.6%, respectively. We could predict two-thirds of the EGFR gene copy number alterations for the lymph node metastasis group or the recurrence tumor group from analysis of the primary tumor. For OSCC patients, in whom the lymph nodes or recurrence tumors were unavailable for EGFR gene copy number analysis, studies of the primary tumor could provide part of the EGFR clonal information to predict metastatic or recurrent lesions.

## Additional file

Additional file 1: Questionnaire for OSCC patients. The questionnaire used in this study includes detailed information on general demographic data, current and past cigarette smoking, alcohol consumption, areca-quid (AQ) chewing, and a history of family disease. (DOCX 35 kb)

## Abbreviations

AQ: Areca-quid
DFS: Disease-free survival
FISH: Fluorescence in situ hybridization
LA/Poly: Low amplification/polysomy
NSCLC: Non-small cell lung cancer
OS: Overall survival
OSCC: Oral squamous cell carcinoma
SPT: Secondary primary tumor
