# Supplementary material for: EGFR copy number alterations in primary tumors, metastatic lymph nodes, and recurrent and multiple primary tumors in oral cavity squamous cell carcinoma
Source: BMC Cancer. 2017 Aug 30;17:592. doi: 10.1186/s12885-017-3586-9 (PMC5576106; doi:10.1186/s12885-017-3586-9)
Supplement: Additional file 1: — Questionnaire for OSCC patients. The questionnaire used in this study includes detailed information on general demographic data, current and past cigarette smoking, alcohol consumption, areca-quid (AQ) chewing, and a history of family disease. (DOCX 35 kb) [file 12885_2017_3586_MOESM1_ESM.docx]

**Questionnaire for Oral Cavity Cancer Patients**

**General information:**

Gender: _____________ (Male/ Female)

Date of birth: ___________________(Ex. 2001/01/01)

Date of diagnosis: ___________________(Ex. 2001/01/01)

**Habits of carcinogen exposure:**

1. Cigarette: ________ (Yes/No)

Amount: _______ ppd，Duration_____ years，Filter _______ (Yes/No)

2. Alcohol: ________ (Yes/No)

Amount: _______ (cups)，Type: ______ (Whisky/Red wine/Beer, etc.)

Frequency _______ (Daily/Weekly/Monthly)，Duration_____ years

3. Areca quid: ________ (Yes/No)

Amount: _______ /day，Duration_____ years

**Family history:**

1. Any family members suffered from malignancies: _____ (Yes/No)

If yes, please specify the relationship and type of malignancy:

Relationship: ______________ (ex. Father/Mother/Brother/Sister/Son/Daughter)

Type of malignancy: ____________________
